# Exosomes in Cancer: Circulating Immune-Related Biomarkers

**DOI:** 10.1155/2019/1628029

**Published:** 2019-12-11

**Authors:** Alicja Głuszko, Mirosław J. Szczepański, Nils Ludwig, Shafaq M. Mirza, Wioletta Olejarz

**Affiliations:** ^1^Chair and Department of Biochemistry, Medical University of Warsaw, Poland; ^2^Department of Pathology, University of Pittsburgh School of Medicine, Pittsburgh, PA 15213, USA; ^3^University of Pittsburgh Cancer Institute, Hillman Cancer Center, Pittsburgh, PA 15213, USA; ^4^Department of Biochemistry and Pharmacogenomics, Faculty of Pharmacy, Medical University of Warsaw, Poland; ^5^Laboratory of Biochemistry and Clinical Chemistry at the Centre for Preclinical Research, Medical University of Warsaw, Poland

## Abstract

Exosomes, the smallest vesicles (30–100 nm) among multivesicular bodies, are released by all body cells including tumor cells. The cargo they transfer plays an important role in intercellular communication. Tumor-derived exosomes (TEXs) maintain interactions between cancer cells and the microenvironment. Emerging evidence suggests that tumor cells release a large number of exosomes, which may not only influence proximal tumor cells and stromal cells in the local microenvironment but can also exert systemic effects as they are circulating in the blood. TEXs have been shown to boost tumor growth promote progression and metastatic spread via suppression or modification of the immune response towards cancer cells, regulation of tumor neo-angiogenesis, pre-metastatic niche formation, and therapy resistance. In addition, recent studies in patients with cancer suggest that TEXs could serve as tumor biomarker reflecting partially the genetic and molecular content of the parent cancer cell (i.e., as a so-called “liquid biopsy”). Furthermore, recent studies have demonstrated that exosomes may have immunotherapeutic applications, or can act as a drug delivery system for targeted therapies with drugs and biomolecules.

## 1. Introduction

**Figure 1:**
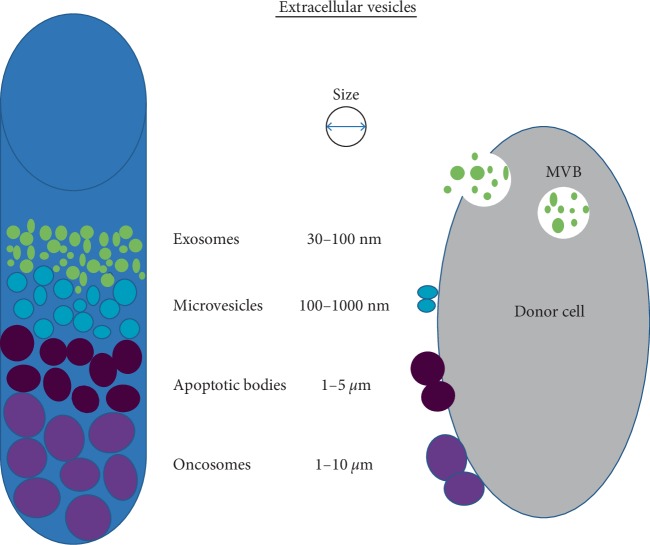
An illustration presents the differences in extracellular vesicles (EV) differentiated on the basis of size, released by donor cells, both normal as well as tumor cells (2, 3, modif.).

Exosomes, which are a subset of extracellular vesicles (EV) are small, lipid bilayer membrane vesicles (30–100 nm) derived from the luminal membrane of multivesicular bodies (MVBs), which are constitutively released by fusion with the cell membrane (Figure 1) [1–3]. Exosomes carry a complex biomolecular cargo which includes proteins, peptides, lipids, and nucleic acids. Interestingly, the genetic cargo of exosomes, such as mRNA and miRNA can be translated or can regulate gene expression in the recipient or target cells [4]. Exosomes are discharged from many cell types including red blood cells, platelets, lymphocytes, dendritic cells, and cancer cells [5]. A growing body of evidence emphasizes their role in pathophysiological processes including malignancies, infectious diseases, autoimmune diseases, metabolic diseases, cardiovascular diseases, and neurodegenerative disorders. Current research focusses on the tumor-promoting roles of exosomes. Tumor growth, angiogenesis, extracellular matrix remodeling, metastasis, and immune surveillance have been shown to be promoted by exosomes [6, 7]. Studies of plasma-derived exosomes in patients with malignancies indicate that tumor-derived exosomes (TEXs) reflect in part the molecular and genetic content of the parent tumor cells. In addition, the molecular cargo of immune cell-derived exosomes (IEX) might serve as biomarkers of immune dysfunction, which facilitates tumor escape. The individual analysis of plasma-derived TEX and IEX by fractionation is expected to identify biomarkers relevant to the tumor as well as determine the immune competence of the cancer patient [8].

## 2. Biogenesis of Exosomes

In contrast to microvesicles, which are secreted by budding from the cell membrane, exosomes show a complex multistep biogenesis, which can be dependent on or independent of the endosomal sorting complex required for transport (ESCRT). ESCRT is a multimolecular machinery, which is recruited to the endosomal membrane for the orchestration of the individual steps of exosome biogenesis [9]. Alternatively, an ESCRT-independent pathway has been described. For this pathway, the specific lipid composition of the endosomal membrane was considered to be of relevance for the exosome biogenesis. Following the formation of MVBs, which is a crucial step in exosome biogenesis, Rab GTPases govern their degradation as well as their secretion [10, 11]. The final release of exosomes occurs upon the fusion of MVBs with the cellular plasma membrane, a process which is probably mediated, at least in part, by soluble N-ethylmaleimide-sensitive factor attachment protein receptors (SNAREs) [12]. Exosome secretion is regulated by various factors, which include mainly environmental changes [13]. Furthermore, the release of exosomes is an effective mechanism, by which cells regulate their internal stress states and modulate the extracellular environment [14]. In the tumor microenvironment, cancer cells are exposed to stressful conditions, such as hypoxia, chemotherapeutics, irradiation, starvation, or other patient-specific factors. One reaction to this microenvironment is the accelerated release of exosomes by cancer cells. Especially hypoxia is an important environmental factor for the secretion of exosomes, since cells produce higher levels of exosomes under hypoxia and the oxygenation status of the parent cells is reflected in the cargo composition of the released exosomes [15]. Exosome-mediated signaling in cancers is thus influenced by various stressful situations [16], and can promote cancer development through interaction between the cancer cells and the neighboring stroma, the stimulation of proliferative and angiogenic signaling, the progression of immune repression, and the initiation of premetastatic niches [17, 18]. Exosomes bind at the cell surface of the recipient cells through specific receptors or undergo internalization by endocytosis or micropinocytosis, following fusion with internal sections [19, 20]. They play crucial roles in most physiological processes in tissues and organs [21].

## 3. Exosome Protein Cargo Secretion and Uptake

**Figure 2:**
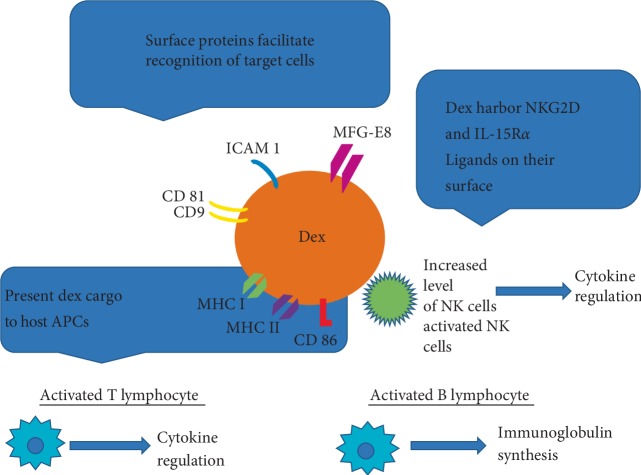
An illustration presents the profiles of exosomes derived from dendritic cells (DEXs). These exosomes may be immunostimulatory. DEXs were found to possess a variety of surface membrane proteins, which could potentially stimulate CD8+ and CD4+ T cells; allow for effective targeting and docking to recipient cells; which are postulated to participate in exosome/acceptor cell interactions, or activate NK (Natural Killer) cells leading to cytokine regulation and immunoglobulin synthesis (26, modif.).

**Figure 3:**
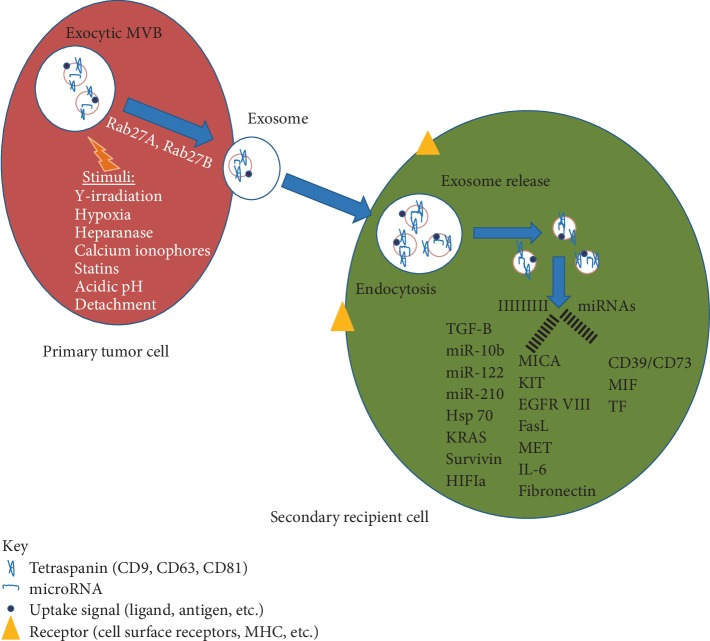
A simplified illustration of some of the different components of exosome cargo (depending on the tumor cell of origin) to highlight the functional changes, that may be induced in recipient cell resulting in tumor progression and metastasis (27, modif.).

Exosomes are defined by a complex cargo consisting out of proteins, lipids, and nucleic acids. Since many different cell types secrete exosomes (e.g., immune cells, epithelial cells, endothelial cells, and cancer cells) and the cargo composition highly varies dependent on the cell of origin, exosomes can be involved in diverse physiological and pathological processes, such as antigen presentation, tissue repair, intercellular cross-talk, and tumor progression [22–24]. The protein content of exosomes can be used for the characterization of exosomes. Some proteins can be found in exosomes regardless of their origin and are considered as “exosome markers”. These include TSG101, Alix, Rab GTPases, heat shock proteins (HSP70, HSP90), integrins, tetraspanins (CD9, CD63, CD81), and MHC class II proteins. Additionally, exosomes can contain genetic material such as mRNA, long noncoding RNA (lncRNA), and microRNA (miRNA) [25] (Figures 2 and 3). The cargo of exosomes mostly reflects the parent cells; however, the composition of exosomes can be different from the cells of their origin due to the selective sorting of cargo into exosomes [26–28]. Exosomes bind at the cell surface of the recipient cells through specific receptors or undergo internalization by various different pathways [19, 20]. The basic mechanism is endocytosis, whereby the extracellular vesicle is engulfed by the recipient cell [29]. There are several mechanisms of endocytosis, such as clathrin-dependent or -independent, caveolin-mediated, macropinocytosis, phagocytosis, and lipid raft-mediated endocytosis. The utilization of those pathways highly depends on the exosomal cargo, the type of recipient cell, and the composition of the cell surface. Additionally, the microenvironment plays a crucial role in exosome internalization [30]. Another mechanism for exosomes uptake is fusion, whereby the exosomes fuse with the membranes of the recipient cell and the content of the vesicle is released into the cell. Fusion efficiency is enhanced in an acidic microenvironment [15, 31].

## 4. The Biological Function of Exosomes in Cancer

### 4.1. Tumorigenesis and Promotion of Tumor Growth

Exosomes are key mediators of intercellular communications between local and distant parts of the body; in cancer, they provide a means to sustain tumor growth and aggressiveness [32]. Numerous results have established tumor-derived exosomes and their specific cargo as key regulators of tumor neo-angiogenesis [33], therapy resistance [34], and pre-metastatic niche formation [35]. In addition, TEXs are important mediators of tumor immune escape and regulation of T cell homeostasis [36–41]. Exosomes released by tumor cells express immunosuppressive molecules such as Fas-ligand (FasL) [42], tumor necrosis factor-related apoptosis-inducing ligand (TRAIL), programmed death-ligand 1 (PD-L1), and interleukin 10 (IL-10), neo-angiogenesis factors, as well as microenvironment conditioning factors, e.g., transforming growth factor *β1* (TGF-*β*1), prostaglandin E2 (PGE2), and ectoenzymes engaged in the adenosine pathway (CD39 and CD73) [43–45]. TEXs carry a variety of molecules within their cargo Figure 2 [26]. Among the most characterized, are small (17–24 nucleotides), one-stranded, noncoding fragments of RNA called microRNA (miRNA), which regulate many biological cell functions such as proliferation, differentiation, migration, angiogenesis, apoptosis, or tumorigenesis [46–53]. Regulating gene transcription, exosomal miRNA trigger mostly pro-cancerous alterations, which we can observe as decreased expression of suppressor genes and intensification of inflammatory processes or drug resistance. This role assigned them the name oncomiRNAs [51, 54–56]. Correlated with tumorigenesis, oncomiRNAs include miR-21, miR-223, miR-210, miR-92a, miR-105, miR- 23b, miR-224, miR-921, and miR29 [57]. Studies of TEX in patients with a variety of cancers attempt to correlate the levels of these exosomes and tumor progression. The data show that the promotion of tumor growth is accompanied by an increased expression level of genes encoding miR-21, necessary for proliferation of tumor cells, their migration, and enhanced invasiveness [58]. Other oncomiRNAs involved in the process of invading noncancerous cells within the tumor microenvironment include miR-223 and miR-105, which were observed in breast cancer cell lines SKBR3, MDA-MB-231 and MCF-10A, MDA-MB-231, respectively. MiR-105 decreased expression of ZO-1 gene encoding Tight Junction Protein-1 in endothelial cells providing increased permeability and as a consequence, facilitated metastasis [59, 60]. Mi-RNAs from the family of let-7 regulate expression of proto-oncogenes RAS and HMGA2, which gives them, likewise miR-23b, miR-224, and miR-921, invasive potential [61, 62]. Promotion of tumor growth and metastasis may also follow stimulation of proinflammatory cytokine release, through pathways other than regulation of gene expression. In this scheme, miR-21 and miR-29 play the role of toll-like-receptor ligands. Activation of TLR receptors present on immune cells stimulates NF-*κ*B (Nuclear Factor kappa-light-chain-enhancer of activated B cells), and as a consequence generates an inflammatory state in the tumor microenvironment [63].

### 4.2. Tumor Angiogenesis and Metastasis

Angiogenesis is another component of tumor formation, that the TEX's miRNAs are involved in. Studies on different cancer cell lines demonstrates that the release of TEXs carrying miR-210, miR17-92, and especially miR92a, (which provide a pro-angiogenic effect), is elevated. It was illustrated that miR-92a inhibits the synthesis of integrin *α*5 in endothelial cells, which increased cell junctions and migration potential [64, 65]. TEXs can modify normal cells pheno- and genotype, not only by microRNA, but also oncogene EGFRvIII transfer or by enhancing mRNA expression for pro-angiogenic factors such as VEGF (vascular endothelial growth factor), HGF (hepatocyte growth factor), and IL-8, thereby enabling tumor cell adhesion to endothelium, resulting in metastasis [66–68]. Adherence and simultaneous stimulation of fibroblastic stroma to release pro-angiogenic factors are supported by ICAM-1 (intercellular adhesion molecule-1) and VCAM (vascular adhesion molecule), carried also by TEXs [69]. Moreover, some TEXs influence angiogenesis directly by VEGF/VEGFR—vascular endothelial growth factor/receptor cargo or pro-angiogenic lipids like S1P [70, 71]. The invasive and migrative potential of tumor cells, apart from adhesive proteins' presence and pro-angiogenic factors, also depends on their ability to degrade extracellular matrix proteins. Studies showed that some TEXs carry metalloproteinases like MMP2, MMP9, MT1-MMP, their inductor EMMPRIN (extracellular matrix metalloprotease inducer), or inactive zymogenes as well as urokinase-type plasminogen activator (u*PA), *which activates zymogenes, and finally, Cathepsin *β*, which is activated at low pH, characteristic for the tumor environment. These factors are responsible for the degradation of collagen, fibrinolysis, and destruction of the extracellular matrix, respectively [71–74].

### 4.3. Tumor Immune Escape

Tumor development can take place unrepressed, because immune surveillance is diminished, as a result of modulation by TEXs [75]. Lack of aberration recognition is possible by different pathways, such as immune cell modulation by exosomal miRNAs. In a variety of cancer types overexpression of miR-9 is observed, which inhibits MHC I (Major Histocompatibility Complex) protein expression on tumor cells; or miR-222 and miR-339, thus decreasing gene expression encoding ICAM-1 adhesive protein, leading to the dysfunctional recognition of tumor cells by the immune system [75–77]. Moreover, among suppressor miRNAs released by TEXs in the tumor microenvironment, we can list miR-23b, miR-224, miR-921, and the let-7 family [57]. Another strategy of tumor immune escape is through the defective recognition of tumor cells by Tc lymphocytes and NK cells, caused by a loss of antigens as a consequence of increased TEXs excretion [78]. In the same way, tumor cells can lose caspase 3, the executive enzyme of apoptosis, probably leading to enhanced tumor cell survival [79]. TEXs also affect effector and regulatory lymphocytes in contrasting methods. Studies showed TEX can inhibit the proliferation of Tc cells by decreasing IL-2 level, essential to this process. On the other hand, TEX carrying FasL, TRAIL, or galectin 9 molecules, can stimulate apoptosis of Tc [80, 81]. In contrast, TEXs promote the expansion and activity of inhibitors of the immune response (Treg and MDSC) [68, 82]. It was observed that melanoma- and colorectal cancer-derived exosomes, after incubation with monocytes, promote their differentiation into MDSC, and moreover stimulate them to release transforming growth factor *β* (TGF-*β*), inhibiting proliferation of T lymphocytes, and as a result negatively influence differentiation into dendritic cells [83, 84].

### 4.4. Drug Resistance

The TEXs role is assigned not only to tumor growth promotion, but also to participation in drug resistance. It is summarized, that TEXs can act directly by effluxing chemotherapeutic drugs from tumor cells or indirectly by carrying glycoprotein P, essential in the multidrug resistance process, to cells that are sensitive to cancer therapies [75, 85]. Some studies demonstrate TEXs take part in doxorubicin or cisplatin elimination from ovarian tumor resistant cells [82, 86]. Disturbance of immunotherapies against cancer is maintained by recognizing and binding tumor-reactive antibodies by TEXs, and carrying tumor antigens instead of antigens present on tumor cells, which dramatically reduces Ab-dependent anticancer mechanisms [87, 88].

## 5. Exosomes as Cancer Therapeutic Targets

Exosomes present in plasma, represent a slight percent of its total proteins composition, but contain diversified molecular profiles, strictly depending on the cell they originated from. It correlates with the type of cancer and even with the progression stage, which gives rise to the potential use for them as biomarkers [89–92]. As exosomes can be used in cancer therapies, their properties have to be taken into consideration. Namely, it was observed, that exosomes derived from immune cells are resistant to lysis dependent on complement factor activation and their cargo of mRNA and microRNA is protected from degradation by RNAases, which makes them good candidates for use as a vaccine [93, 94]. Moreover, an abundance of dendritic cell-derived exosomes, (DEXs) surface proteins such as tetraspanin family, ICAM-1, MFG-E8, facilitates their interaction with target cells [95, 96]. Preclinical studies showed DEXs have the potential to activate TCD4+ and TCD8+ in melanoma and nonsmall cell lung cancer patients and present safety and feasibility of application [97–99]. It has also been reported that ascites-derived exosomes (ADE) and granulocyte-macrophage colony-stimulating factor (GM-CSF) induced strong anti-cancer T cell response in patients with advanced stages of colorectal cancer [100]. DEXs may also present their cargo to antigen presenting cells (APC), enhancing immune response and triggering NKG2D ligands, in turn stimulating NK cells, which is reflected in increased NK levels in melanoma patients in a clinical trial [97, 101–103]. At present, current research results support us with promising data regarding vaccination utilizing DEX. The advantages of DEX anti-cancer therapy include feasibility, safety, stability, and effectiveness [104, 105].

## 6. Conclusion

The research on exosomes is at its naissance. It is already apparent, that we have to acknowledge the new intercellular communication and the cell function regulation systems, which are established by these nanovesicles. The process of their biogenesis, secretion, and uptake grants them an important physiological function, greatly diversified by the variety of transported molecules on their surface or as cargo. The characterization of the molecula composition of exosomes, particularly those that are tumor-derived, needs to be extended following the advancement of technologies for their isolation and precise separation methods from normal cell-derived exosomes, in order to achieve the foundation for a new diagnostic tool. Exosome-mediated bioactive molecules transfer, including nucleic acids, proteins, and lipids, influencing homeostatic changes within pheno-, genotypic adaptation and immune evasion, represent an undoubtedly huge potential for clinical and therapeutic advances. Extensive research is needed to fully understand and incorporate the application of this powerful source of new data provided by the exosomes.

## Abbreviations

Rab GTPasae: Member of Ras superfamily of monomeric G proteins
EV: Extracellular vesicles
TSG101: Tumor susceptibility gene 101
HMGA2: High-mobility group AT-hook 2
S1P: Site-1 protease.

## References

[1] Colombo M., Raposo G., Théry C. (2014). Biogenesis, secretion, and intercellular interactions of exosomes and other extracellular vesicles. *Annual Review of Cell and Developmental Biology*.

[2] Bence G., Szabó T. G., Pásztói M. (2011). Membrane vesicles, current state-of-the-art: emerging role of extracellular vesicles. * Cellular and Molecular Life Sciences*.

[3] Minciacchi V. R., Zijlstra A., Rubin M. A., Di Vizio D. (2017). Extracellular vesicles for liquid biopsy in prostate cancer: where are we and where are we headed?. *Prostate Cancer and Prostatic Diseases*.

[4] Peterson M. F., Otoc N., Sethi J. K., Gupta A., Antes T. J. (2015). Integrated systems for exosome investigation. *Methods*.

[5] Tkach M., Théry C. (2016). Communication by extracellular vesicles: where we are and where we need to go. *Cell*.

[6] Kalluri R. (2016). The biology and function of exosomes in cancer. *Journal of Clinical Investigation*.

[7] Zhang X., Yuan X., Shi H., Wu L., Qian H., Xu W. (2015). Exosomes in cancer: small particle, big player. *Journal of Hematology & Oncology*.

[8] Whiteside T. L. (2018). The emerging role of plasma exosomes in diagnosis, prognosis and therapies of patients with cancer. *Contemporary Oncology (Poznan, Poland)*.

[9] Williams R. L., Urbé S. (2007). The emerging shape of the ESCRT machinery. *Nature Reviews Molecular Cell Biology*.

[10] Ostrowski M., Carmo N. B., Krumeich S. (2010). Rab27a and Rab27b control different steps of the exosome secretion pathway. *Nature Cell Biology*.

[11] Robbins P. D., Morelli A. E. (2014). Regulation of immune responses by extracellular vesicles. *Nature Reviews Immunology*.

[12] Fader C. M., Sánchez D. G., Mestre M. B., Colombo M. I. (2009). TI-VAMP/VAMP7 and VAMP3/cellubrevin: two v-SNARE proteins involved in specific steps of the autophagy/multivesicular body pathways pathways. *Biochimica et Biophysica Acta (BBA) – Molecular Cell Research*.

[13] Kowal J., Tkach M., Théry C. (2014). Biogenesis and secretion of exosomes. *Current Opinion in Cell Biology*.

[14] Villarroya-Beltri C., Baixauli F., Gutiérrez-Vázquez C., Sánchez-Madrid F., Mittelbrunn M. (2014). Sorting it out: regulation of exosome loading. * Seminars in Cancer Biology*.

[15] Parolini I., Federici C., Raggi C. (2009). Microenvironmental pH is a key factor for exosome traffic in tumor cells. *Journal of Biological Chemistry*.

[16] De Jong O. G., Verhaar M. C., Chen Y. (2012). Cellular stress conditions are reflected in the protein and RNA content of endothelial cell-derived exosomes. *Journal of Extracellular Vesicles*.

[17] Wieckowski E. U., Visus C., Szajnik M., Szczepanski M. J., Storkus W. J., Whiteside T. L. (2009). Tumor-derived microvesicles promote regulatory T cell expansion and induce apoptosis in tumor-reactive activated CD8+ T lymphocytes. *Journal of Immunology*.

[18] Szajnik M., Czystowska M., Szczepanski M. J., Mandapathil M., Whiteside T. L. (2010). Tumor-derived microvesicles induce, expand and up-regulate biological activities of human regulatory T cells (Treg). *PLoS One*.

[19] Segura E., Guérin C., Hogg N., Amigorena S., Théry C. (2007). CD8+ dendritic cells use LFA-1 to capture MHC-peptide complexes from exosomes in vivo. *Journal of Immunology*.

[20] Fitzner D., Schnaars M., van Rossum D. (2011). Selective transfer of exosomes from oligodendrocytes to microglia by macropinocytosis. *Journal of Cell Science*.

[21] Kim Y.-S., Ahn J.-S., Kim S., Kim H.-J., Kim S.-H., Kang J.-S. (2018). The potential theragnostic (diagnostic+therapeutic) application of exosomes in diverse biomedical fields. *The Korean Journal of Physiology & Pharmacology*.

[22] Thery C., Boussac M., Véron P. (2001). Proteomic analysis of dendritic cell-derived exosomes: a secreted subcellular compartment distinct from apoptotic vesicles. *The Journal of Immunology*.

[23] Valadi H., Ekström K., Bossios A., Sjöstrand M., Lee J. J., Lötvall J. O. (2007). Exosome-mediated transfer of mRNAs and microRNAs is a novel mechanism of genetic exchange between cells. *Nature Cell Biology*.

[24] Lai R. C., Arslan F., Lee M. M. (2010). Exosome secreted by MSC reduces myocardial ischemia/reperfusion injury. *Stem Cell Research*.

[25] Melo S. A., Sugimoto H., O’Connell J. T (2014). Cancer exosomes perform cell-independent microRNA biogenesis and promote tumorigenesis. *Cancer Cell*.

[26] Pitt J. M., André F., Amigorena S. (2016). Dendritic cell-derived exosomes for cancer therapy. *Journal of Clinical Investigation*.

[27] Whiteside T. L. (2016). Tumor-derived exosomes and their role in cancer progression. *Advances in Clinical Chemistry*.

[28] Crescitelli R., Lässer C., Szabó T. G. (2013). Distinct RNA profiles in subpopulations of extracellular vesicles: apoptotic bodies, microvesicles and exosomes. *Journal of Extracellular Vesicles*.

[29] Mulcahy L. A., Pink R. C., Carter D. R. F. (2014). Routes and mechanisms of extracellular vesicle uptake. *Journal of Extracellular Vesicles*.

[30] Stahl A.-L., Johansson K., Mossberg M., Kahn R., Karpman D. (2017). Exosomes and microvesicles in normal physiology, pathophysiology, and renal diseases. *Pediatric Nephrology*.

[31] Guo W., Gao Y., Li N. (2017). Exosomes: new players in cancer (review). *Oncology Reports*.

[32] Fernandez-Messina L., Gutiérrez-Vázquez C., Rivas-García E., Sánchez-Madrid F., de la Fuente H. (2015). Immunomodulatory role of microRNAs transferred by extracellular vesicles. *Biology of the Cell*.

[33] Umezu T., Tadokoro H., Azuma K., Yoshizawa S., Ohyashiki K., Ohyashiki J. H. (2014). Exosomal miR-135b shed from hypoxic multiple myeloma cells enhances angiogenesis by targeting factor-inhibiting HIF-1. *Blood*.

[34] Gong J., Jaiswal R., Mathys J-m, Combes V., Grau G., Bebawy M. (2012). Microparticles and their emerging role in cancer multidrug resistance. *Cancer Treatment Reviews*.

[35] Peinado H., Alečković M., Lavotshkin S. (2012). Melanoma exosomes educate bone marrow progenitor cells toward a pro-metastatic phenotype through MET. *Nature Medicine*.

[36] Abusamra A. J., Zhong Z., Zheng X. (2005). Tumor exosomes expressing Fas ligand mediate CD8+ T-cell apoptosis. *Blood Cells, Molecules, and Diseases*.

[37] Kim J. W., Wieckowski E., Taylor D. D., Reichert T. E., Watkins S., Whiteside T. L. (2005). Fas ligand-positive membranous vesicles isolated from sera of patients with oral cancer induce apoptosis of activated T lymphocytes. *Clinical Cancer Research*.

[38] Bergmann C., Strauss L., Wieckowski E. (2009). Tumor-derived microvesicles in sera of patients with head and neck cancer and their role in tumor progression. * Head & Neck*.

[39] Martinez-Lorenzo M. J., Anel A., Alava M. A. (2004). The human melanoma cell line MelJuSo secretes bioactive FasL and APO2L/TRAIL on the surface of microvesicles. possible contribution to tumor counterattack. *Experimental Cell Research*.

[40] Huber V., Fais S., Iero M. (2005). Human colorectal cancer cells induce T-cell death through release of proapoptotic microvesicles: role in immune escape. *Gastroenterology*.

[41] Qu J.-L., Qu X.-J., Qu J.-L. (2009). The role of cbl family of ubiquitin ligases in gastric cancer exosome-induced apoptosis of jurkat T cells. *Acta Oncologica*.

[42] Van Parijs L., Abbas A. K. (1996). Role of Fas-mediated cell death in the regulation of immune responses. *Current Opinion in Immunology*.

[43] Schuler P. J., Saze Z. (2014). Human CD4+ CD39+ regulatory T cells produce adenosine upon co-expression of surface CD73 or contact with CD73+ exosomes or CD73+ cells. *Clinical and Experimental Immunolog*.

[44] Boyiadzis M., Whiteside T. L. (2017). The emerging roles of tumor-derived exosomes in hematological malignancies. *Leukemia*.

[45] Whiteside T. L. (2016). Exosomes and tumor-mediated immune suppression. *Journal of Clinical Investigation*.

[46] Bartel D. P. (2004). MicroRNAs: genomics, biogenesis, mechanism, and function. *Cell*.

[47] Lin S., Gregory R. I. (2015). MicroRNA biogenesis pathways in cancer. *Nature Reviews Cancer*.

[48] Kota J., Chivukula R. R., O'Donnell K. A. (2009). Therapeutic microRNA delivery suppresses tumorigenesis in a murine liver cancer model. *Cell*.

[49] Tay Y., Zhang J., Thomson A. M., Lim B., Rigoutsos I. (2008). MicroRNAs to Nanog, Oct4 and Sox2 coding regions modulate embryonic stem cell differentiation. *Nature*.

[50] Wang Y., Lee C. G. L. (2009). MicroRNA and cancer–focus on apoptosis. *Journal of Cellular and Molecular Medicine*.

[51] Zhang B., Pan X., Cobb G. P., Anderson T. A. (2007). MicroRNAs as oncogenes and tumor suppressors. *Developmental Biology*.

[52] Landskroner-Eiger S., Moneke I., Sessa W. C. (2013). MiRNAs as modulators of angiogenesis. *Cold Spring Harbor Perspectives in Medicine*.

[53] Tang Y., Lin Y., Li C. (2015). MicroRNA-720 promotes in vitro cell migration by targeting Rab35 expression in cervical cancer cells. *Cell & Bioscience*.

[54] Liu J., Zheng M., Tang Y.-L., Liang X.-H., Yang Q. (2011). MicroRNAs, an active and versatile group in cancers. *International Journal of Oral Science*.

[55] Falcone G., Felsani A., D’Agnano I. (2015). Signaling by exosomal microRNAs in cancer. *Journal of Experimental & Clinical Cancer Research*.

[56] Esquela-Kerscher A., Slack F. J. (2006). Oncomirs — microRNAs with a role in cancer. *Nature Reviews Cancer*.

[57] Wesolowska A., Piwocka K. (2017). Exosomal microRNAs as a part of the cell-cell communication in cancer. *Postepy Biochemistry*.

[58] Lu Z., Liu M., Stribinskis V. (2008). MicroRNA-21 promotes cell transformation by targeting the programmed cell death 4 gene. *Oncogene*.

[59] Zhou W., Fong M. Y, Min Y. (2014). Cancer-secreted miR-105 destroys vascular endothelial barriers to promote metastasis. *Cancer Cell*.

[60] Yang M., Chen J., Su F. (2011). Microvesicles secreted by macrophages shuttle invasion-potentiating microRNAs into breast cancer cells. *Molecular Cancer*.

[61] Ohshima K., Inoue K., Fujiwara A. (2010). Let-7 microRNA family is selectively secreted into the extracellular environment via exosomes in a metastatic gastric cancer cell line. *PLoS One*.

[62] Ostenfeld M. S., Jeppesen D. K., Laurberg J. R. (2014). Cellular disposal of miR23b by RAB27-dependent exosome release is linked to acquisition of metastatic properties. *Cancer Research*.

[63] Fabbri M., Paone A., Calore F., Galli R., Croce C. M. (2013). A new role for microRNAs, as ligands of toll-like receptors. *RNA Biology*.

[64] Umezu T., Ohyashiki K., Kuroda M., Ohyashiki J. H. (2013). Leukemia cell to endothelial cell communication via exosomal miRNAs. *Oncogene*.

[65] Grange C., Tapparo M., Collino F. (2011). Microvesicles released from human renal cancer stem cells stimulate angiogenesis and formation of lung premetastatic niche. *Cancer Research*.

[66] Al-Nedawi K., Meehan B., Micallef J. (2008). Intercellular transfer of the oncogenic receptor EGFRvIII by microvesicles derived from tumour cells. *Nature Cell Biology*.

[67] Janowska-Wieczorek A., Wysoczynski M., Kijowski J. (2005). Microvesicles derived from activated platelets induce metastasis and angiogenesis in lung cancer. *International Journal of Cancer*.

[68] Skog J., Würdinger T., van Rijn S. (2008). Glioblastoma microvesicles transport RNA and proteins that promote tumour growth and provide diagnostic biomarkers. *Nature Cell Biology*.

[69] Wubbolts R., Leckie R. S., Veenhuizen P. T. M. (2003). Proteomic and biochemical analyses of human B cell-derived exosomes. Potential implications for their function and multivesicular body formation. *Journal of Biological Chemistry*.

[70] Distler J. H., Pisetsky D. S., Huber L. C., Kalden J. R., Gay S., Distler O. (2005). Microparticles as regulators of inflammation: novel players of cellular crosstalk in the rheumatic diseases. *Arthritis & Rheumatism*.

[71] Baj-Krzyworzeka M., Szatanek R., Węglarczyk K. (2006). Tumour-derived microvesicles carry several surface determinants and mRNA of tumour cells and transfer some of these determinants to monocytes. *Cancer Immunology, Immunotherapy*.

[72] Dolo V., D'Ascenzo S., Violini S. (1999). Matrix-degrading proteinases are shed in membrane vesicles by ovarian cancer cells in vivo and in vitro. *Clinical & Experimental Metastasis*.

[73] Hakulinen J., Sankkila L., Sugiyama N., Lehti K., Keski-Oja J. (2008). Secretion of active membrane type 1 matrix metalloproteinase (MMP-14) into extracellular space in microvesicular exosomes. *Journal of Cellular Biochemistry*.

[74] Giusti I., D’Ascenzo S., Millimaggi D. (2008). Cathepsin B mediates the pH-dependent proinvasive activity of tumor-shed microvesicles. *Neoplasia*.

[75] Filipazzi P., Bürdek M., Villa A., Rivoltini L., Huber V. (2012). Recent advances on the role of tumor exosomes in immunosuppression and disease progression. *Seminars in Cancer Biology*.

[76] Gao F., Zhao Z.-L., Zhao W.-T. (2013). miR-9 modulates the expression of interferon-regulated genes and MHC class I molecules in human nasopharyngeal carcinoma cells. *Biochemical and Biophysical Research Communications*.

[77] Ueda R., Kohanbash G., Sasaki K. (2009). Dicer-regulated microRNAs 222 and 339 promote resistance of cancer cells to cytotoxic T-lymphocytes by down-regulation of ICAM-1. *Proceedings of the National Academy of Sciences*.

[78] Cocucci E., Racchetti G., Meldolesi J. (2009). Shedding microvesicles: artefacts no more. *Trends in Cell Biology*.

[79] Boing A. N., Hau C. M., Sturk A., Nieuwland R. (2008). Platelet microparticles contain active caspase 3. *Platelets*.

[80] Andre F., Schartz N. E. C., Movassagh M. (2002). Malignant effusions and immunogenic tumour-derived exosomes. *The Lancet*.

[81] Andreola G., Rivoltini L., Castelli C. (2002). Induction of lymphocyte apoptosis by tumor cell secretion of FasL-bearing microvesicles. *The Journal of Experimental Medicine*.

[82] Safaei R., Larson B. J., Cheng T. C. (2005). Abnormal lysosomal trafficking and enhanced exosomal export of cisplatin in drug- resistant human ovarian carcinoma cells. *Molecular Cancer Therapeutics*.

[83] Colino J., Snapper C. M. (2006). Exosomes from bone marrow dendritic cells pulsed with diphtheria toxoid preferentially induce type 1 antigen-specific IgG responses in naive recipients in the absence of free antigen. *The Journal of Immunology*.

[84] Valenti R., Huber V., Iero M., Filipazzi P., Parmiani G., Rivoltini L. (2007). Tumor-released microvesicles as vehicles of immunosuppression. *Cancer Research*.

[85] Ambudkar S. V., Sauna Z. E., Gottesman M. M., Szakacs G. (2005). A novel way to spread drug resistance in tumor cells: functional intercellular transfer of P-glycoprotein (ABCB1). *Trends in Pharmacological Sciences*.

[86] Shedden K., Xie X. T., Chandaroy P., Chang Y. T., Rosania G. R. (2003). Expulsion of small molecules in vesicles shed by cancer cells: association with gene expression and chemosensitivity profiles. *Cancer Research*.

[87] Battke C., Ruiss R., Welsch U. (2011). Tumour exosomes inhibit binding of tumour-reactive antibodies to tumour cells and reduce ADCC. *Cancer Immunology, Immunotherapy*.

[88] Ciravolo V., Huber V., Ghedini G. C. (2012). Potential role of HER2-overexpressing exosomes in countering trastuzumab-based therapy. *Journal of Cellular Physiology*.

[89] Baran J., Baj-Krzyworzeka M., Weglarczyk K. (2010). Circulating tumour-derived microvesicles in plasma of gastric cancer patients. *Cancer Immunology, Immunotherapy*.

[90] Chaput N., Théry C. (2011). Exosomes: immune properties and potential clinical implementations. *Seminars in Immunopathology*.

[91] Smalley D. M., Ley K. (2008). Plasma-derived microparticles for biomarker discovery. *Clinical Laboratory*.

[92] Welton J. L., Khanna S., Giles P. J. (2010). Proteomics analysis of bladder cancer exosomes. *Molecular & Cellular Proteomics*.

[93] Clayton A., Harris C. L, Court J., Mason M. D., Morgan B. P. (2003). Antigen-presenting cell exosomes are protected from complement-mediated lysis by expression of CD55 and CD59. *European Journal of Immunology*.

[94] Trajkovic K., Hsu C., Chiantia S. (2008). Ceramide triggers budding of exosome vesicles into multivesicular endosomes. *Science*.

[95] Gabrilovich D. I., Ciernik I. F., Carbone D. P. (1996). Dendritic cells in antitumor immune responses. I. Defective antigen presentation in tumor-bearing hosts. *Cellular Immunology*.

[96] Thery C., Ostrowski M., Segura E. (2009). Membrane vesicles as conveyors of immune responses. *Nature Reviews Immunology*.

[97] Escudier B., Dorval T., Chaput N. (2005). Vaccination of metastatic melanoma patients with autologous dendritic cell (DC) derived-exosomes: results of the first phase I clinical trial. *Journal of Translational Medicine*.

[98] Morse M. A., Garst J., Osada T. (2005). A phase I study of dexosome immunotherapy in patients with advanced non-small cell lung cancer. *Journal of Translational Medicine*.

[99] Tan A., De La Peña H., Seifalian A. M. (2010). The application of exosomes as a nanoscale cancer vaccine. *International Journal of Nanomedicine*.

[100] Dai S., Wei D., Wu Z. (2008). Phase I clinical trial of autologous ascites-derived exosomes combined with GM-CSF for colorectal cancer. *Molecular Therapy*.

[101] Chaput N., Flament C., Viaud S. (2006). Dendritic cell derived-exosomes: biology and clinical implementations. *Journal of Leukocyte Biology*.

[102] Quah B. J., O'Neill H. C. (2005). The immunogenicity of dendritic cell-derived exosomes. *Blood Cells, Molecules, and Diseases*.

[103] Zitvogel L., Regnault A., Lozier A. (1998). Eradication of established murine tumors using a novel cell-free vaccine: dendritic cell-derived exosomes. *Nature Medicine*.

[104] Andre F., Escudier B., Angevin E., Tursz T., Zitvogel L. (2004). Exosomes for cancer immunotherapy. *Annals of Oncology*.

[105] Zhang B., Yin Y., Lai R. C., Lim S. K. (2014). Immunotherapeutic potential of extracellular vesicles. *Frontiers in Immunology*.

